# Assessment and Clinical Relevance of Serum IL-19 Levels in Psoriasis and Atopic Dermatitis Using a Sensitive and Specific Novel Immunoassay

**DOI:** 10.1038/s41598-019-41609-z

**Published:** 2019-03-26

**Authors:** Robert J. Konrad, Richard E. Higgs, George H. Rodgers, Wenyu Ming, Yue-Wei Qian, Nicoletta Bivi, Justin K. Mack, Robert W. Siegel, Brian J. Nickoloff

**Affiliations:** Lilly Research Laboratories, Eli Lilly and Company, Indianapolis, IN 46285 USA

## Abstract

Because development of reliable biomarkers in psoriasis and atopic dermatitis has lagged behind therapeutic progress, we created a blood-based test to fill the void in objective methods available for dermatological assessments. Our novel interleukin-19 (IL-19) immunoassay was initially tested to determine concentrations of IL-19 serum levels, then correlated with the psoriasis activity and severity index (PASI) in psoriasis, and the eczema area and severity index (EASI) in atopic dermatitis. Not only was IL-19 increased in psoriasis and correlated to PASI, but ixekizumab administration led to rapid, sustained IL-19 decreases to normal levels, with decreases at 2-weeks correlating with PASI improvement at 16-weeks. IL-19 increased upon ixekizumab withdraw, prior to relapse, and decreased following re-treatment. In baricitinib- and etanercept-treated psoriasis patients, IL-19 decreases also correlated with improvement. Many patients with limited skin disease, including genital psoriasis and psoriatic arthritis patients, also had increased IL-19, which was reduced to normal levels upon ixekizumab treatment, correlating with PASI improvement. We also measured IL-19 in baricitinib-treated atopic dermatitis patients. In atopic dermatitis, IL-19 was significantly elevated, correlated with EASI scores, and decreased with skin improvement. Therefore, measurement of serum IL-19 provides clinicians with an objective disease-activity assessment tool for psoriasis and atopic dermatitis patients.

## Introduction

Psoriasis is a chronic inflammatory skin disease mediated by a dysregulated immune system characterized by red, thickened skin and an angiogenic tissue response to stress, injury, and infection^1,2^. It has a high global prevalence (approximately 125 million^2^) and life-long nature, with disease manifestations above and below the skin surface. Unfortunately, there has not been as much progress in a disease scoring system as there has been in development of drugs to treat the disease itself^3–7^. Currently, plaque type psoriasis is typically measured using the naked eye for calculating the overall body surface area (BSA) involvement alone, or combined with the degree of erythema, thickness, and scaling of lesions (psoriasis area and severity index, PASI), or using the static Physician’s Global Assessment (sPGA). These visual methods to assess disease activity may not be linear with limited skin involvement.

Reproducible, quantitative methods to determine severity are clearly needed to assess more accurately disease activity and response to therapy, as more psoriasis patients are being treated with systemic agents^8^. It is also unclear if limited skin involvement truly reflects mild disease or if absence of evident skin involvement, including erythema, reflects complete resolution, especially in patients of color. While psoriasis patients are typically classified as having moderate-to-severe disease when BSA is greater than 10% and PASI is greater than 10, the lack of a reliable test method for inflammatory burden has precluded addressing whether patients with so-called mild skin involvement actually have significantly less overall disease activity^3^.

These translational gaps in contemporary medical practice prompted us to search for a novel, objective biomarker assay for monitoring disease activity across a spectrum of skin involvement using sera from psoriasis patients characterized for baseline and post-treatment clinical disease activity. We focused on interleukin 19 (IL-19), a member of the IL-10 cytokine family implicated in inflammation, vascularization, and tissue remodeling^9^. In this study, we developed a sensitive and specific novel immunoassay for IL-19 to reveal that baseline circulating IL-19 levels correlate with skin involvement in psoriasis. This novel IL-19 biomarker assay allowed us to demonstrate for the first time that decreases in serum IL-19 levels precede clinical responses in psoriasis when the therapeutic anti-IL-17A antibody ixekizumab is administered. In addition, the research presented here is the first to show that increases in circulating IL-19 observed following withdrawal of ixekizumab precede skin relapses. We also demonstrate that our IL-19 assay has similar applicability for psoriasis patients treated with tumor necrosis factor (TNF)α inhibitors such as etanercept, or Janus associated kinase (JAK) 1/2 inhibitors such as baricitinib. Importantly, our research shows that the IL-19 results obtained in psoriasis are replicable in atopic dermatitis, a common and relapsing inflammatory skin disease (with less defined skin lesions than psoriatic plaques) affecting a wide range of age groups with high global prevalence^10–12^.

While others have suggested that multiple molecular signatures may be required for clinical relevance in psoriasis^13^, we demonstrate that IL-19 alone may serve as a single, serum-based biomarker to assess disease severity. Our findings indicate that a serum IL-19 assay for disease activity could provide to clinicians managing psoriasis patients an important tool to complement the therapeutic advances being made in treating this chronic, immune-mediated, skin disease^14^.

## Materials and Methods

### Human samples

From the Eli Lilly Research Blood Donor program with consent, 36 serum samples from healthy volunteers were obtained. For clinical studies, we obtained samples after patients gave permission for their serum to be collected for exploratory analyses. Patient samples were de-identified to protect privacy, shipped on dry ice, and stored at −80 °C. All clinical studies were conducted in accordance with ethical principles of the Declaration of Helsinki and Good Clinical Practice guidelines and were approved by the institutional review board or ethics committee of each participating site (including Chesapeake IRB, Copernicus Group IRB, Ethikkommission der Ärztekammer, Komisja Bioetyczna przy Okregowej Izbie Lekarskiej w Krakowie, Research Review Board Inc., Schulman Associates IRB, and Western IRB). All patients provided written informed consent.

### Ixekizumab phase 2 psoriasis serum samples

We obtained samples from 112 patients enrolled in a phase 2 psoriasis study (NCT01107457, first posted to ClinicalTrials.gov on April 21, 2010) for ixekizumab^15^. Patients were assigned to five treatment groups and were administered either placebo or 10, 25, 75, or 150 mg ixekizumab at 0, 2, 4, 8, 12, and 16 weeks. Samples were collected at baseline and after 2 weeks and 16 weeks of treatment, and at additional time points (for patients that entered the study extension).

### Ixekizumab and etanercept phase 3 psoriasis serum samples

Samples were collected from patients with psoriasis enrolled in a phase 3 ixekizumab study (UNCOVER-2, NCT01597245, first posted to ClinicalTrials.gov on May 14, 2012), which included an etanercept active comparator arm^16^. Samples were collected at baseline and after 1, 4, and 12 weeks of treatment with either etanercept (50 mg biweekly) or ixekizumab (loading dose of 160 mg followed by 80 mg every two weeks [Q2W]). For ixekizumab, 31 patients were selected at random from a subgroup of patients who were sPGA 0, 1 responders (representing clear or almost clear skin) at week 12 and then switched to placebo after induction. Additional samples were also collected at later time points. Because ixekizumab-treated patients had greatly improved PASI responses compared to etanercept patients, stratified random sampling of etanercept-treated patients (35 etanercept patients) was performed to match the week 12 PASI distribution of the ixekizumab patients. In addition, a stratified random sampling of etanercept-treated patients was performed (164 etanercept patients) to match the overall etanercept PASI distribution. This set of 164 etanercept patients included the subset of 35 patients matched to the ixekizumab response.

### Ixekizumab-treated psoriatic arthritis patients

Baseline, 4-week, and 12-week treatment samples were collected from 309 patients with psoriatic arthritis throughout the induction period of a phase 3 study (SPIRIT-P1, NCT02349295, first posted to ClinicalTrials.gov on January 28, 2015), during which patients were administered either placebo or ixekizumab (a loading dose of 160 mg followed by 80 mg Q2W or 80 mg every four weeks [Q4W])^17^. This patient cohort with psoriatic arthritis had significantly less skin involvement as assessed by BSA and PASI compared to the moderate-to-severe psoriasis patients^16,17^.

### Ixekizumab-treated genital psoriasis patients

Baseline, 2-week, 4-week, and 12-week treatment samples were collected from 120 patients with genital psoriasis throughout the induction period of a phase 3b study (NCT02718898, first posted to ClinicalTrials.gov on March 24, 2016), during which patients were administered either placebo or ixekizumab (160 mg loading dose followed by 80 mg Q2W) for 12 weeks^18^.

### Baricitinib-treated psoriasis patients

We obtained samples from 270 patients from a phase 2 psoriasis study (NCT01490632, first posted to ClinicalTrials.gov on December 13, 2011) for the JAK1/2 inhibitor baricitinib^19^. Patients were randomly assigned to five groups and were administered either placebo or 2, 4, 8, or 10 mg of baricitinib once daily (QD) for 12 weeks. Samples were collected at baseline and after 12 weeks of treatment.

### Atopic dermatitis patients

Samples were obtained from 124 atopic dermatitis patients enrolled in a phase 2 study (NCT02576938, first posted to ClinicalTrials.gov on October 15, 2015) for baricitinib^20^. Patients in this study received topical corticosteroids as a standardization period for 4 weeks, and were then randomly assigned to placebo, or baricitinib 2 mg or 4 mg QD for 16 weeks. During the treatment period, all patients remained on topical corticosteroids. Samples for IL-19 were obtained at baseline (following the 4 weeks of lead-in topical corticosteroids), and after 4 weeks and 16 weeks of treatment.

### Rheumatoid arthritis and ulcerative colitis patients

Baseline samples were analyzed from 122 patients with rheumatoid arthritis from a phase 2 rheumatoid arthritis study (NCT00966875, first posted to ClinicalTrials.gov on August 27, 2009)^21^ and 117 patients with ulcerative colitis from a phase 2 ulcerative colitis study (NCT02589665, first posted to ClinicalTrials.gov on October 28, 2015).

### Statistical Methods

The MSD software was used for fitting the IL-19 calibration curves. One-way analysis of variance (ANOVA) models were fit using log_10_ transformed analyte concentrations using the aov function of R version 3.5.0^22^. All reported correlations were estimated using the non-parametric, Spearman’s, method using the function cor.test of R version 3.5.0^22^. Repeated measures pharmacokinetic analyses were conducted with SAS® software version 9.4 using PROC MIXED with an unstructured variance-covariance matrix and log_10_ transformed analyte concentration as the response^23^. Receiver operating characteristic (ROC) areas were estimated using the roc() function from the R package pROC^24^. ROC area confidence intervals were estimated using 1000 bootstrap samplings. P-values less than 0.05 were considered statistically significant. The upper limit of normal was calculated based on a sampling of 36 healthy volunteers. All graphics were generated using the R package ggplot2^25^.

Additional methods and results for the expression and purification of human recombinant IL-19, the generation, purification, selection, and labeling of murine monoclonal anti-human IL-19 antibodies, and the IL-19 immunoassay are included in the Supplementary File and Fig. S1.

## Results

**Figure 1:**
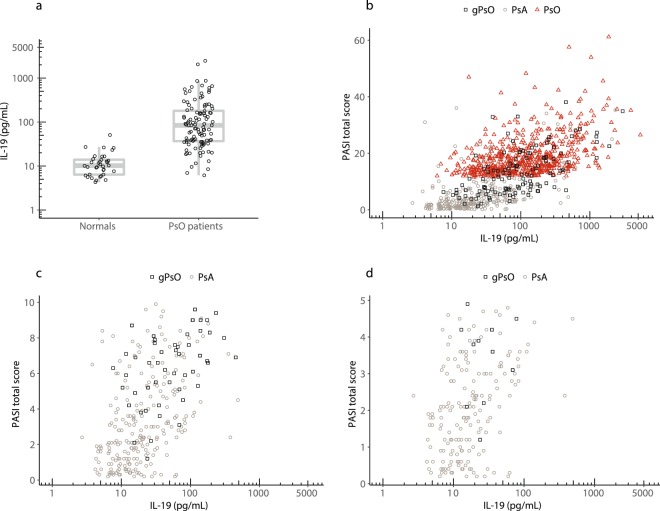
Baseline IL-19 levels are markedly increased in psoriasis and are highly correlated with PASI scores in psoriasis, genital psoriasis, and psoriatic arthritis patients. (**a**) The normal donor IL-19 geometric mean was 11 pg/mL, while in psoriasis (PsO) patients at baseline, the IL-19 geometric mean was 87 pg/mL (*p* < 0.0001). **(b**) Baseline IL-19 levels were measured in 556 patients with psoriasis (PsO), 120 patients with genital psoriasis (gPsO), and 293 patients with psoriatic arthritis (PsA). IL-19 was highly correlated with PASI (Spearman’s r = 0.64, *p* < 0.0001). **(c**) Same as Fig. 1b but restricted to PASI < 10 (Spearman’s r = 0.53, *p* < 0.0001). **(d**) Same as Fig. 1b but restricted to PASI < 5 (Spearman’s r = 0.39, *p* < 0.0001).

To determine if circulating IL-19 was elevated in patients with psoriasis and/or correlated with PASI scores, serum IL-19 was first measured in 36 healthy subjects (Fig. 1a). Levels were detectable in all normal samples with values ranging from 2–51 pg/mL, a geometric mean of 11 pg/mL, and an upper limit of normal of 21 pg/mL (robust 95^th^ percentile). In contrast, serum IL-19 concentrations measured in baseline samples from patients with psoriasis enrolled in a phase 2 ixekizumab psoriasis study were markedly elevated (geometric mean of 87 pg/mL, *p* < 0.0001).

To determine if IL-19 levels in psoriasis and psoriatic arthritis patients were correlated with PASI scores, we measured IL-19 in baseline samples of 556 patients with psoriasis (from phase 2 and phase 3 ixekizumab studies and a phase 2 baricitinib study), 293 patients with psoriatic arthritis (from a phase 3 ixekizumab study), and 120 patients with genital psoriasis (from a phase 3b ixekizumab study), and compared IL-19 results to PASI scores. The inclusion of psoriatic arthritis patients and genital psoriasis patients provided an opportunity to evaluate IL-19 levels across a range of PASI scores, including patients with limited BSA (<10%). Baseline IL-19 levels in these 969 total patients were markedly elevated and were highly correlated with PASI scores (Fig. 1b, Spearman’s r = 0.64, *p* < 0.0001).

Interestingly, even at relatively low PASI scores of less than 10 (Fig. 1c) or 5 (Fig. 1d), there was still significant correlation of IL-19 with PASI (Spearman’s r-values of 0.53 and 0.39, respectively, both *p* < 0.0001). In addition, some patients with relatively low PASI scores had elevated serum IL-19 levels, suggesting that even small, localized skin disease could be associated with increased circulating IL-19. Importantly, the Spearman’s correlation of 0.64 between baseline PASI and IL-19 is likely an upper limit for correlation based on the correlation of PASI versus PASI performed by different raters^26^.

**Figure 2:**
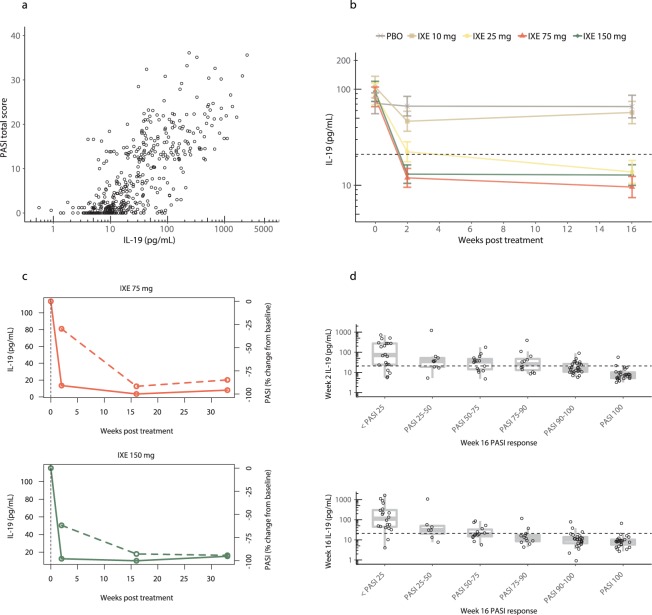
Circulating IL-19 levels serve as a leading indicator of PASI improvement in psoriasis patients treated with ixekizumab. **(a**) We obtained samples from 112 psoriasis patients enrolled in a phase 2 study (NCT01107457) for ixekizumab^15^. Patients were assigned to five treatment groups and were administered placebo or 10, 25, 75, or 150 mg ixekizumab at 0, 2, 4, 8, 12, and 16 weeks. Circulating IL-19 concentrations were measured at baseline and after ≥16 weeks of treatment. All data from baseline and after 16 weeks of treatment are shown. Collectively, serum IL-19 levels were highly correlated with PASI (Spearman’s r = 0.74, *p* < 0.0001). (**b**) IL-19 was measured in psoriasis patients from Fig. 2a at baseline and over 16 weeks of treatment. Data are least square means ± standard error of the mean estimated from a mixed effects model using an unstructured covariance matrix. Model fitting was performed using log_10_ transformed IL-19 concentrations. The dashed horizontal line indicates the upper limit of normal (21 pg/mL). Week 2 comparisons of ixekizumab (IXE) to placebo (PBO): 10 mg (*p* = 0.28), 25 mg (*p* = 0.0015), 75 mg (*p* < 0.0001), 150 mg (*p* < 0.0001); Week 16 comparisons: 10 mg (*p* = 0.71), 25 mg (*p* < 0.0001), 75 mg (*p* < 0.0001), 150 mg (*p* < 0.0001). **(c**) Representative patient profiles from Fig. 2b for IL-19 (solid) and PASI percent change from baseline (dashed). A patient treated with 75 mg of ixekizumab (IXE) is shown on top and a patient treated with 150 mg of ixekizumab is shown on bottom. The dashed vertical line indicates the baseline time point. In each case, IL-19 decreases prior to PASI improvement. **(d**) Serum IL-19 levels in psoriasis patients from Fig. 2b after 2 weeks (top) and 16 weeks (bottom) of placebo or doses of ixekizumab are plotted versus PASI at 16 weeks. The dashed horizontal line indicates the upper limit of normal. PASI 100 improvements at 16 weeks were preceded by IL-19 reductions to near normal levels after 2 weeks of treatment. Top panel one-way ANOVA (*p* < 0.0001). Bottom panel one-way ANOVA (*p* < 0.0001).

We next measured IL-19 concentrations in phase 2 psoriasis patients at baseline and after 16 weeks of treatment with placebo or various doses of ixekizumab and compared the results with PASI scores. Serum IL-19 levels, at baseline and endpoint, in patients with psoriasis were again highly correlated with PASI (Fig. 2a, Spearman’s r = 0.74, *p* < 0.0001). In this study, patients were administered placebo or four different doses of ixekizumab (10, 25, 75, or 150 mg) at 0, 2 and 4 weeks and then Q4W through 16 weeks. As Fig. 2b shows, placebo treatment resulted in no significant change in IL-19 levels. In contrast, ixekizumab was associated with a dramatic dose-dependent lowering of IL-19 levels at 2 weeks that was sustained throughout 16 weeks of treatment (*p* < 0.0001 for 75 and 150 mg doses versus placebo at 2 and 16 weeks).

The highest doses of ixekizumab administered (75 and 150 mg) were associated with normal mean IL-19 levels at 2 weeks. Figure 2c compares the IL-19 levels to PASI change from baseline in representative patients treated with either 75 or 150 mg of ixekizumab. Interestingly, IL-19 concentrations markedly decreased prior to improvement in the patients’ PASI scores. This pattern was observed in the majority of patients treated with therapeutic doses of ixekizumab (data not shown). In almost all cases, a precipitous drop in IL-19 levels at 2 weeks preceded a dramatic improvement in PASI at 16 weeks. These dosing regimens were similar to those used in the phase 3 ixekizumab studies (160 mg loading dose followed by 80 mg Q2W or Q4W).

In light of these intriguing findings, we further examined the temporal relationship between the decrease in IL-19 levels and the change from baseline in PASI at a trial population level. To perform this analysis, the absolute levels of IL-19 at the 2-week and 16-week time points were compared to the percent change from baseline in PASI after 16 weeks. Figure 2d shows the results of these analyses. The 16-week IL-19 level was highly correlated with PASI improvement at 16 weeks (one-way ANOVA *p* < 0.0001). Importantly, at the 2-week time point, absolute IL-19 levels were also highly correlated with PASI improvement observed after 16 weeks (one-way ANOVA *p* < 0.0001).

As Fig. 2d illustrates, most patients who had a reduction in IL-19 levels to less than 21 pg/mL (the upper limit of normal) had dramatic improvement in their PASI scores, with the majority of these patients achieving PASI 90. On the other hand, there was much less consistent PASI response in the group of patients (many in the placebo and 10 mg ixekizumab groups) who did not achieve this degree of IL-19 lowering. Many of these patients failed to achieve PASI 75. The area under the ROC curve (plot of true positive rate versus false positive rate) was used to assess predictive performance of IL-19, with an area of 1 indicating perfect classification and an area of 0.5 indicating random classification. Using IL-19 as an indicator of week 16 PASI 75 response yielded ROC curve areas of 0.77 and 0.85 for week 2 and week 16, respectively. Similarly, for PASI 90 responses, ROC curve areas were 0.80 and 0.82 for week 2 and week 16, respectively.

**Figure 3:**
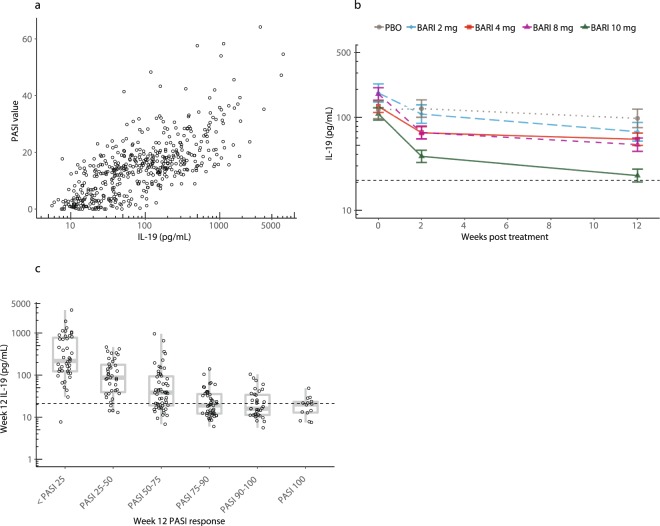
IL-19 is highly correlated with PASI in psoriasis patients treated with the JAK1/2 inhibitor baricitinib. (**a**) We obtained samples from 270 patients enrolled in a phase 2 psoriasis study (NCT01490632) for the JAK1/2 inhibitor baricitinib^19^. Patients were administered placebo or 2, 4, 8, or 10 mg of baricitinib QD for 12 weeks. Samples were collected at baseline and after 12 weeks of treatment. Circulating IL-19 was measured at baseline and after 12 weeks of treatment with baricitinib (2, 4, 8, or 10 mg QD) or placebo in patients with psoriasis. All data from baseline and after 12 weeks of treatment are shown. Collectively, serum IL-19 levels were highly correlated with PASI (Spearman’s r = 0.72, *p* < 0.0001). **(b**) Serum IL-19 was measured in psoriasis patients at baseline and after 12 weeks of treatment with placebo (PBO) or baricitinib (BARI) (2, 4, 8, or 10 mg QD). Data plotted are least square means ± standard error of the mean estimated from a mixed effects model using an unstructured covariance matrix. Model fitting was performed using log_10_ transformed IL-19 concentrations. The dashed horizontal line indicates the upper limit of normal (21 pg/mL). Week 12 comparisons of baricitinib to placebo: 2 mg (*p* = 0.31), 4 mg (*p* = 0.063), 8 mg (*p* = 0.022), and 10 mg (*p* < 0.0001). (**c**) Serum IL-19 levels in psoriasis patients after 12 weeks of treatment with placebo or baricitinib (2, 4, 8, or 10 mg QD) are plotted versus PASI at 12 weeks. The dashed horizontal line indicates the upper limit of normal. PASI >75 improvements at 12 weeks were correlated with reduction of circulating IL-19 concentrations. One-way ANOVA (*p* < 0.0001).

Next, we measured IL-19 levels in patients with psoriasis in a phase 2 trial (investigating the JAK1/2 inhibitor baricitinib) at baseline and after 12 weeks of treatment with placebo or various doses of baricitinib and compared IL-19 levels with PASI scores (Fig. 3a). Serum IL-19 levels in these patients were highly correlated with PASI (Spearman’s r = 0.72, *p* < 0.0001). Baseline IL-19 levels were again markedly elevated. As anticipated, placebo treatment resulted in no significant change in IL-19 over the 12-week time period. In contrast, baricitinib treatment was associated with a dose-dependent lowering of IL-19 levels (*p*-values of 0.31, 0.063, 0.022, and < 0.0001 for 2, 4, 8, and 10 mg doses, respectively), although the decreases were not as robust as those observed with ixekizumab; and even the highest dose of baricitinib (10 mg QD) did not normalize IL-19 concentrations (Fig. 3b).

After analyzing these data, we examined the relationship between the decrease in IL-19 levels and the change from baseline in PASI at a trial population level by comparing the absolute levels of IL-19 at the 12-week time point to the percent change from baseline in PASI at 12 weeks. Figure 3c shows the results of these analyses, in which the 12-week IL-19 level was highly correlated with PASI improvement at the same time point (one-way ANOVA *p* < 0.0001). Patients who had reductions in their IL-19 levels to less than the upper limit of normal tended to have the most dramatic improvement in their PASI scores. Alternatively, there was much less consistent PASI response in patients who did not achieve this degree of IL-19 lowering, with many of these patients failing to achieve PASI 75.

**Figure 4:**
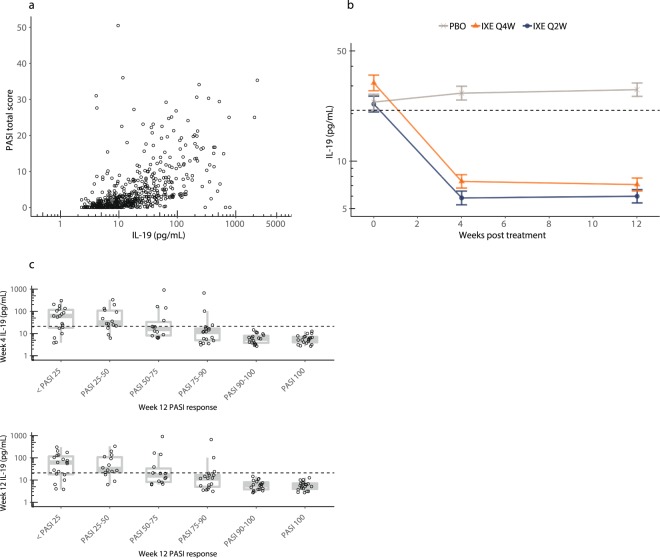
Serum IL-19 is highly correlated with PASI in psoriatic arthritis, and ixekizumab treatment causes a rapid, sustained decrease in IL-19, with levels serving as a leading indicator of PASI improvement. (**a**) Baseline, 4-week, and 12-week treatment samples were collected from 309 psoriatic arthritis patients throughout the induction period of a phase 3 study (SPIRIT-P1, NCT02349295), during which patients were administered placebo or ixekizumab (a loading dose of 160 mg followed by 80 mg Q2W or 80 mg every four weeks [Q4W]) for 12 weeks^17^. Patients enrolled in this study had no minimum baseline PASI. Circulating IL-19 was measured at baseline and after 12 weeks of treatment with ixekizumab (80 mg Q2W or 80 mg Q4W) or placebo. All data from baseline and after 12 weeks of treatment are shown. Collectively, serum IL-19 results were highly correlated with PASI (Spearman’s r = 0.66, *p* < 0.0001). (**b**) Circulating IL-19 was measured in psoriatic arthritis patients at baseline and during 12 weeks of treatment with placebo (PBO) or ixekizumab (IXE) (80 mg Q2W or 80 mg Q4W). Data are least square means ± standard error of the mean estimated from a mixed effects model using an unstructured covariance matrix. Model fitting was performed using log_10_ transformed IL-19 concentrations. The dashed horizontal line indicates the upper limit of normal (21 pg/mL). Week 4 comparisons of ixekizumab to placebo: 80 mg Q2W (*p* < 0.0001), 80 mg Q4W (*p* < 0.0001); Week 12 comparisons: 80 mg Q2W (*p* < 0.0001), 80 mg Q4W (*p* < 0.0001). (**c**) Serum IL-19 levels in psoriatic arthritis patients with baseline PASI ≥5 after 4 weeks (top) of placebo or ixekizumab (80 mg Q2W or 80 mg Q4W) treatment, or 12 weeks (bottom) of placebo or ixekizumab (80 mg Q2W or 80 mg Q4W) treatment are plotted versus PASI at 12 weeks. The dashed horizontal line indicates the upper limit of normal. PASI 100 improvements at 12 weeks were preceded by reduction of IL-19 to near normal levels after 4 weeks of treatment. Top panel one-way ANOVA (*p* < 0.0001). Bottom panel one-way ANOVA (*p* < 0.0001).

After obtaining these results in psoriasis patients, we next measured IL-19 concentrations in patients with psoriatic arthritis enrolled in the phase 3 ixekizumab trial, SPIRIT-P1, at baseline and after 12 weeks of treatment with ixekizumab and compared the results with PASI scores. Circulating IL-19 levels in patients with psoriatic arthritis were highly correlated with PASI scores (Fig. 4a, Spearman’s r = 0.66, *p* < 0.0001). Baseline IL-19 levels in patients with psoriatic arthritis were increased compared to normal, although not to the level seen in patients with moderate-to-severe psoriasis (Fig. 4b). As expected, placebo treatment resulted in no significant change in IL-19 levels. In contrast, administration of ixekizumab (loading dose of 160 mg followed by 80 mg Q2W or 80 mg Q4W) dramatically lowered IL-19 levels to within the normal range after 4 weeks of treatment, and this was sustained throughout 12 weeks of treatment (all *p*-values < 0.0001 for ixekizumab Q2W and Q4W versus placebo at 4 weeks and 12 weeks). Similar to what was observed in psoriasis, PASI 100 improvements at 12 weeks were correlated with reduction of circulating IL-19 to normal levels at 4 weeks (one-way ANOVA *p* < 0.0001), with the majority of the poor PASI responders being in the placebo group (Fig. 4c).

Interestingly, in patients with psoriatic arthritis, there was no correlation of IL-19 with any marker of joint involvement, including the health assessment questionnaire-disability index (HAQ-DI) or patient’s assessment of joint pain (data not shown). These results suggested that IL-19 was a specific marker of skin involvement, rather than a non-specific marker of inflammation like C-reactive protein. We confirmed this by measuring baseline IL-19 levels in rheumatoid arthritis and ulcerative colitis patients and found that IL-19 in these patients was not elevated above the upper limit of normal (geometric means of 17 and 9 pg/mL, respectively, data not shown).

**Figure 5:**
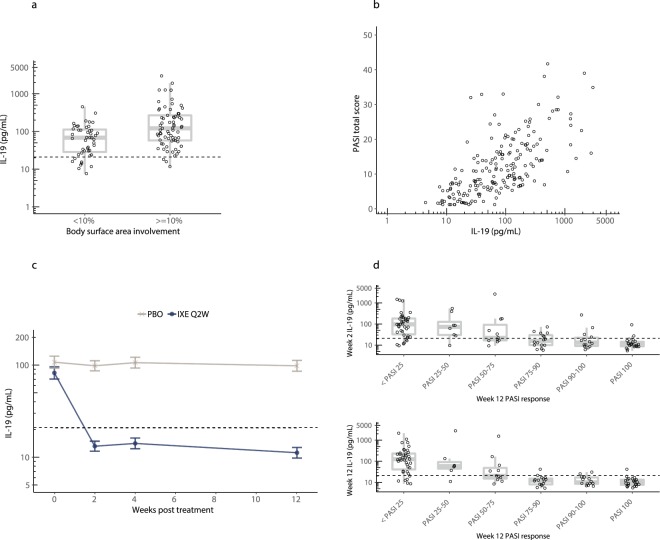
Serum IL-19 is highly correlated with PASI in genital psoriasis, with circulating levels serving as a leading indicator of PASI improvement following ixekizumab treatment. (**a**) Baseline, 2-, 4-, and 12-week treatment samples were collected from 120 genital psoriasis patients throughout the induction period of a phase 3b study (NCT02718898), during which patients were administered placebo or ixekizumab (160 mg loading dose followed by 80 mg Q2W) for 12 weeks^18^. Subjects with moderate‐to‐severe genital psoriasis defined as a baseline static Physician’s Global Assessment of Genitalia (sPGA‐G) score of ≥3, with BSA ≥1% were randomized 1:1 to receive placebo or ixekizumab. At baseline, 47 patients had BSA <10% with an IL-19 geometric mean of 57 pg/mL, while 73 patients had BSA ≥10% with an IL-19 geometric mean of 131 pg/mL. The difference was statistically significant (*p* = 0.0001). (**b**) Circulating IL-19 concentrations were measured at baseline and after 12 weeks of treatment with placebo or ixekizumab 80 mg Q2W in patients with genital psoriasis. All data from baseline and after 12 weeks of treatment are shown. Collectively, serum IL-19 levels were highly correlated with PASI (Spearman’s r = 0.79, *p* < 0.0001). (**c**) IL-19 was measured in genital psoriasis patients at baseline and over 12 weeks of treatment with placebo (PBO) or ixekizumab (IXE) 80 mg Q2W. Data are least square means ± standard error of the mean estimated from a mixed effects model using an unstructured covariance matrix. Model fitting was performed using log_10_ transformed IL-19 concentrations. The dashed horizontal line indicates the upper limit of normal (21 pg/mL). Comparisons between placebo and ixekizumab at weeks 2, 4, and 12 were statistically significant (p < 0.0001). (**d**) Serum IL-19 levels in genital psoriasis patients after 2 (top) and 12 weeks (bottom) of placebo or ixekizumab 80 mg Q2W are plotted versus PASI at 12 weeks. The dashed horizontal line indicates the upper limit of normal. PASI 100 improvements at 12 weeks were preceded by IL-19 reductions to near normal levels after 2 weeks of treatment. One-way ANOVA (*p* < 0.0001) for both panels.

Following these results, we measured IL-19 in genital psoriasis patients enrolled in a phase 3b ixekizumab trial at baseline and after 2, 4, and 12 weeks of treatment with placebo or ixekizumab (160 mg loading dose followed by 80 mg Q2W). Doing so enabled us the ability to study patients with limited BSA involvement. As Fig. 5a shows, even patients with BSA <10% had dramatically elevated baseline IL-19 concentrations (geometric mean of 57 pg/mL, *p* < 0.0001 compared to normals), while patients with BSA ≥10% had even higher IL-19 levels (geometric mean of 131 pg/mL, *p* < 0.0001 compared to normals). IL-19 levels were higher in the ≥10% BSA group compared to the <10% BSA group (*p* = 0.0001).

We next compared baseline and 12-week IL-19 results with PASI scores in patients with genital psoriasis and found them to be highly correlated (Fig. 5b, Spearman’s r = 0.79, *p* < 0.0001). As Fig. 5c demonstrates, ixekizumab reduced IL-19 concentrations to within the normal range after only 2 weeks of treatment, and reductions were sustained throughout 12 weeks, with comparisons between ixekizumab and placebo being statistically significant (*p* < 0.0001) at all post-treatment time points. Similar to what was observed in psoriasis and psoriatic arthritis patients, Fig. 5d shows that PASI 100 improvements at 12 weeks were preceded by IL-19 reductions to normal levels after 2 weeks that were sustained through 12 weeks (one-way ANOVA *p* < 0.0001 for IL-19 at 2 and 12 weeks versus PASI at 12 weeks). Once again, the majority of the poor PASI responders were in the placebo group.

**Figure 6:**
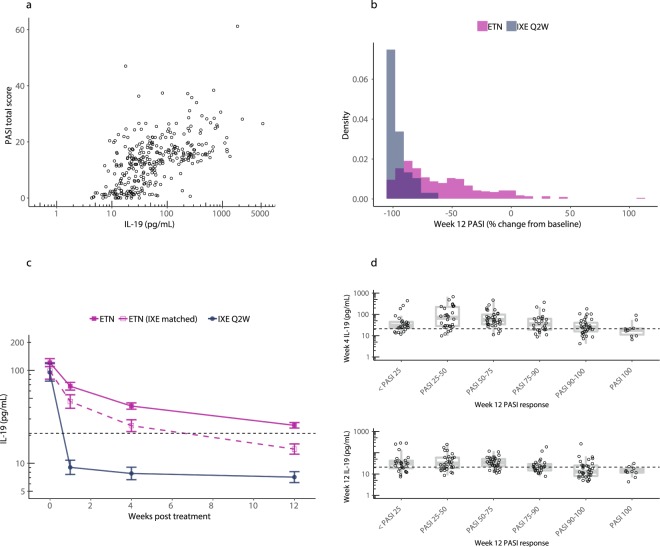
Etanercept decreases circulating IL-19 concentrations in psoriasis patients, but decreases are not as rapid or robust as those observed with ixekizumab. (**a**) Samples were collected from psoriasis patients enrolled in a phase 3 ixekizumab study (UNCOVER-2, NCT01597245), which included an etanercept active comparator arm^16^. Circulating IL-19 was measured at baseline and after 12 weeks of treatment with the TNFα inhibitor etanercept in 163 patients with psoriasis. All data from baseline and after 12 weeks of treatment are shown. Collectively, serum IL-19 results were highly correlated with PASI (Spearman’s r = 0.60, *p* < 0.0001). (**b**) Overlaid histograms were prepared for end-of-induction (week 12) PASI scores for etanercept (ETN) (purple, n = 334, all etanercept patients; 36% of all etanercept patients were sPGA = 0, 1 responders) and ixekizumab (IXE) induction responders who were randomized to placebo and then re-administered ixekizumab upon relapse (blue, n = 94 out of 341 patients initially treated with ixekizumab; 83% of all ixekizumab patients were sPGA = 0, 1 responders). (**c**) Serum IL-19 levels in psoriasis patients were measured at baseline and during 12 weeks of treatment with etanercept (ETN) or ixekizumab (IXE). Data from 164 etanercept patients, 31 ixekizumab responder patients, and 35 of the best-responding etanercept patients (matched for ixekizumab response), were plotted as least square means ± standard error of the mean estimated from a mixed effects model using an unstructured covariance matrix. Model fitting was performed using log_10_ transformed IL-19 concentrations. The dashed horizontal line indicates the upper limit of normal (21 pg/mL). Comparisons between etanercept patients and ixekizumab patients at weeks 1, 4, and 12 were all statistically significant (*p* < 0.0001). (**d**) Serum IL-19 levels after 4 weeks (top) or 12 weeks (bottom) of etanercept treatment are plotted versus PASI at 12 weeks for 164 etanercept patients matched to the overall etanercept PASI distribution. The dashed horizontal line indicates the upper limit of normal. Lower IL-19 levels after treatment with etanercept for 4 weeks or 12 weeks were correlated with improved PASI response at 12 weeks. Top panel one-way ANOVA (*p* < 0.0001). Bottom panel one-way ANOVA (*p* < 0.0001).

To investigate further the role of IL-19 as a biochemical biomarker-based indicator of psoriatic disease activity, we examined the effect of the TNFα inhibitor etanercept. Serum IL-19 levels were measured in patients with psoriasis enrolled in a phase 3 ixekizumab study (UNCOVER-2), which included an etanercept active comparator arm. We identified a subset of 164 etanercept patients using stratified random sampling to match the overall etanercept PASI distribution, measured IL-19 levels in these samples at baseline and after 12 weeks of treatment with etanercept (50 mg biweekly), and compared the results with PASI scores. Circulating IL-19 was again highly correlated with PASI scores (Fig. 6a, Spearman’s r = 0.60, *p* < 0.0001).

Figure 6b shows overlaid histograms for the end-of-induction (week 12) PASI scores for the 334 etanercept patients (36% of etanercept patients were sPGA = 0, 1 responders) and the ixekizumab responders (83% of ixekizumab patients were responders). Because ixekizumab patients had greatly improved PASI responses compared to etanercept patients, stratified random sampling was used to compare the very best responding etanercept-treated patients (35 patients) to a group of randomly selected ixekizumab responders (31 patients) based on the 12-week end-of-induction PASI assessments. Baseline IL-19 levels in the three sets of patients (the 164 etanercept patients, the 31 ixekizumab responders, and the 35 etanercept patients matched for ixekizumab response) were markedly increased compared to normal (Fig. 6c).

Ixekizumab treatment was associated with a lowering of IL-19 levels to within the normal range, which was evident as early as one week and sustained throughout 12 weeks of induction (Fig. 6c). In the subset of the best PASI-responding etanercept patients, etanercept treatment was associated with reduction of IL-19 concentrations to near normal levels; however, decreases were slower and less dramatic than those observed with ixekizumab. In the overall group of 164 etanercept patients, reductions in IL-19 were evident, but to a much lesser degree in terms of both timing and magnitude, with mean IL-19 levels failing to decrease below the upper limit of normal even after 12 weeks. Comparisons of IL-19 between ixekizumab and etanercept patients at weeks 1, 4, and 12 were all statistically significant (*p* < 0.0001). Similar to previous analyses for ixekizumab, we also examined IL-19 levels after 4 weeks and 12 weeks of etanercept treatment and compared them to PASI at 12 weeks for the 164 etanercept-treated patients (Fig. 6d). At both the 4-week and 12-week time points, lower IL-19 levels were correlated with PASI improvement at 12 weeks (one-way ANOVAs of *p* = 0.0001 and *p* < 0.0001, respectively).

**Figure 7:**
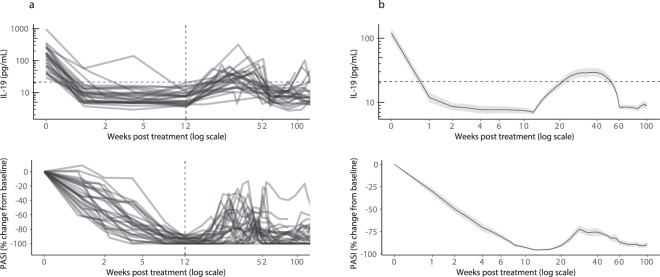
Serum IL-19 levels increase when psoriasis patients treated with ixekizumab are transitioned to placebo and decrease again upon ixekizumab re-treatment. (**a**) Samples were collected from psoriasis patients enrolled in a phase 3 ixekizumab study (UNCOVER-2, NCT01597245)^16^. Serum IL-19 and PASI (percent change from baseline) are shown for responders selected at random who were administered ixekizumab (80 mg Q2W) for 12 weeks and achieved sPGA = 0, 1 (83% of ixekizumab patients were sPGA = 0, 1 responders). After responding, patients were transitioned to placebo and re-treated with ixekizumab when their skin response dissipated. Time course profiles are shown for IL-19 (top) and PASI (bottom) for each individual patient during the initial treatment with ixekizumab, during washout after patients had responded (sPGA = 0, 1), and following re-treatment. Twenty-seven of the 31 ixekizumab patients are included in the analysis (patients with baseline IL-19 levels ≤21 pg/mL were excluded). (**b**) Individual patient time-course profiles from Fig. 7a were linearly interpolated to enable a weekly geometric mean ± standard error for IL-19 and a weekly mean ± standard error for PASI. Interpolated time course profiles are shown for IL-19 (top) and PASI (bottom). The dashed horizontal line indicates the upper limit of normal IL-19 concentrations (21 pg/mL). The rise in IL-19 levels between weeks 12 and 20 is influenced by interpolation because many patients did not have serum samples drawn at every time point when PASI assessments were performed.

Together with the ixekizumab data, these data were strongly suggestive of IL-19 as a leading indicator of skin response after treatment initiation. To determine the role of IL-19 as an indicator of skin response after treatment withdrawal, we examined IL-19 in 31 patients in the phase 3 study (UNCOVER-2) who responded to ixekizumab during induction (sPGA = 0, 1), were switched to placebo, and were then re-treated with ixekizumab after their sPGA score increased to 3 or greater. Figure 7a shows individual time course profiles for IL-19 and PASI for each patient during the initial treatment period, the washout period, and after re-treatment. Figure 7b shows the interpolated time course profiles for IL-19 and PASI for the patients in Fig. 7a. To perform these analyses, individual patient time course profiles were linearly interpolated to estimate weekly geometric means for IL-19 and PASI. The rise in IL-19 levels between weeks 12 and 20 upon discontinuation of ixekizumab is influenced by interpolation because many patients did not have serum samples banked corresponding to every PASI assessment during this timeframe. As these figures demonstrate, the decreases in IL-19 levels observed after initial ixekizumab treatment preceded the subsequent improvements in PASI by approximately 6–8 weeks. Likewise, after withdrawal of ixekizumab, increases in IL-19 preceded worsening PASI by 6–8 weeks.

To assess the utility of IL-19 to indicate PASI 90 response after initiation of therapy, IL-19 and PASI data from 741 patients (treated with ixekizumab, etanercept, or baricitinib) were pooled and analyzed with a logistic regression model to estimate PASI 90 response. This model took into account both baseline and post-baseline (week 12 or 16) IL-19 levels. The ROC area for this model was 0.8, with both the baseline and post-baseline IL-19 levels being statistically significant terms. For comparison, the baseline PASI score and a simulated post-baseline PASI score based on the observed score plus variation consistent with that reported in Gourraud and co-workers^26^ were used to estimate a replicate PASI response. This simulated, replicate PASI response demonstrated an ROC area of 0.7–0.8, comparable to what was observed using the IL-19 data. The logistic regression was repeated using PASI 100 as the response, and in this model only the post-baseline IL-19 level was statistically significant with an ROC area of 0.79.

**Figure 8:**
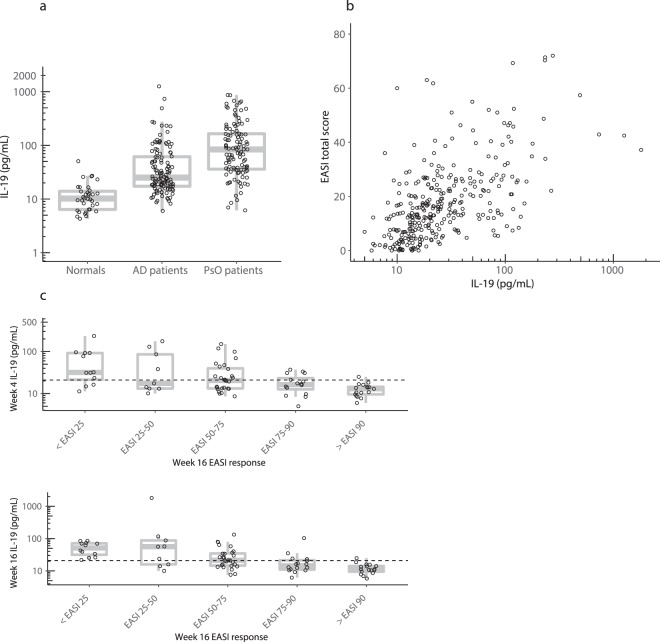
Circulating IL-19 concentrations are increased in atopic dermatitis, correlate with EASI, and serve as a leading indicator of skin improvement. (**a**) Samples were obtained from 123 atopic dermatitis patients enrolled in a phase 2 study (NCT02576938) for baricitinib^20^. Patients received topical corticosteroids as a standardization period for 4 weeks, and were then randomly assigned to placebo, or baricitinib 2 mg or 4 mg QD for 16 weeks. During the 16-week treatment period, all patients remained on topical corticosteroids. Serum samples for IL-19 measurement were obtained at baseline (following the 4 weeks of lead-in topical corticosteroids), and after 4 weeks and 16 weeks of treatment. In atopic dermatitis (AD), the baseline IL-19 geometric mean is 34 pg/mL (*p* < 0.0001 versus normals). Data from normal subjects and psoriasis (PsO) patients are shown for comparison. (**b**) Circulating IL-19 was measured at baseline and during 16 weeks of QD treatment with placebo, 2 mg, or 4 mg of baricitinib in patients with atopic dermatitis. All data from baseline and after 16 weeks of treatment are shown. Collectively, serum IL-19 results were highly correlated with EASI (Spearman’s r = 0.59, *p* < 0.0001). (**c**) Serum IL-19 levels in atopic dermatitis patients after 4 weeks (top) or 16 weeks (bottom) of QD placebo, 2 mg, or 4 mg of baricitinib are plotted versus EASI at 16 weeks. The dashed horizontal line indicates the upper limit of normal (21 pg/mL). EASI improvements of >90 at 16 weeks were preceded by reduction of circulating IL-19 to near normal concentrations after 4 weeks of treatment. Top panel one-way ANOVA (*p* = 0.0002). Bottom panel one-way ANOVA (*p* < 0.0001).

In light of these data, we extended our analyses to atopic dermatitis, a second, common, chronic inflammatory skin disease, by examining IL-19 levels in patients enrolled in a phase 2 study for the JAK1/2 inhibitor baricitinib. In these patients, even after 4 weeks of run-in treatment with topical corticosteroids, baseline serum IL-19 levels were markedly elevated (geometric mean of 34 pg/mL, *p* < 0.0001 compared to normals) (Fig. 8a). Circulating IL-19 measured in these patients at baseline and during 16 weeks of QD treatment with placebo, 2 mg, or 4 mg of baricitinib was correlated (Spearman’s r = 0.59, *p* < 0.0001) with the eczema activity and severity index (EASI) (Fig. 8b). Absolute levels of IL-19 at the 4-week and 16-week time points were compared to the percent change from baseline in EASI after 16 weeks (Fig. 8c). The 16-week IL-19 level was highly correlated with EASI improvement at 16 weeks (one-way ANOVA *p* < 0.0001). In addition, the IL-19 level at 4 weeks was also highly correlated with EASI improvement observed after 16 weeks, indicating that IL-19 was a leading indicator of EASI improvement (one-way ANOVA *p* = 0.0002). Patients whose IL-19 levels decreased to less than the upper limit of normal had dramatic improvement in their EASI scores, with the majority achieving >EASI 90.

## Discussion

An ideal assessment for psoriatic disease severity should be minimally invasive to avoid the scarring that occurs after deep punch biopsies in symptomless or lesional skin and utilize the most objective measurement possible^27–29^. In this study, we demonstrate that serum IL-19 levels objectively reflect disease activity and are correlated with PASI scores across a broad spectrum of psoriatic plaques, including so-called mild, moderate, and severe disease. Our novel IL-19 immunoassay was capable of measuring IL-19 levels in human serum over various time points during administration of therapeutic agents having different mechanisms of action. Using this assay, we show that IL-19 levels are dramatically elevated in psoriasis, genital psoriasis, and psoriatic arthritis, are highly correlated with skin involvement, and function as a leading indicator of skin disease severity during treatment with multiple classes of medications.

Upon treatment of psoriasis, genital psoriasis, and psoriatic arthritis patients with ixekizumab, serum IL-19 decreased prior to PASI improvement. In psoriasis patients, IL-19 increased prior to skin relapse when treatment with ixekizumab was discontinued; and decreased again after re-treatment. In psoriasis patients treated with the JAK1/2 inhibitor baricitinib or the TNFα antagonist etanercept, IL-19 levels were also highly correlated with PASI scores, and decreases correlated with PASI improvement. We also similarly extended the utility of IL-19 to atopic dermatitis, another disease in which it would be desirable to have a minimally invasive objective method to measure disease activity, drug responses, and relapse^28^. We found that in atopic dermatitis, serum IL-19 was markedly elevated, was highly correlated with EASI, and served as a leading biomarker for skin improvement.

While a greater understanding of psoriasis immunopathology has contributed to major advancements in achieving high levels of skin responses rapidly and safely^30^, methods for assessing disease activity have changed very little since the introduction of PASI some forty years ago^7^. In contemplating IL-19 as a biomarker for psoriasis, atopic dermatitis, and possibly other epithelial diseases, we recognized the importance of cellular cross-talk between bone-marrow-derived myeloid cells and epidermal keratinocytes, both of which can produce IL-19. Our results correlating elevated serum levels of IL-19 to disease severity provide additional insight into the complex cytokine network operative in psoriatic plaques^31^. Indeed, the observations that keratinocytes are activated by IL-19, increasing phosphorylated signal transducer and activator of transcription (pSTAT) 3 levels, and that keratinocytes themselves produce IL-19, may explain the important clinical relationships with IL-19 that we identified^31^. Moving below the inflamed, thickened, and scaling skin in psoriatic lesions, there is also distortion of the underlying dermis with prominent vascularity and abundance of monocytes and macrophages, such that both keratinocyte and monocyte-derived IL-19 production can be detected in the blood^32^. Activated macrophages associated with intense skin inflammation contribute to vascular inflammation, and the use of fluodeoxyglucose (FDG)/positron emission tomography (PET)/computerized tomography (CT) imaging in assessing cardiovascular co-morbidities in psoriasis is based on detection of activated macrophages in vascular structures throughout the body^33^.

Greater overall reductions in serum IL-19 levels by ixekizumab compared to etanercept may be explained by the larger induction of IL-19 production in keratinocytes by IL-17 compared to TNFα^34^. Indeed, as pathogenic Th17 cells are highly plastic (with an unstable phenotype), treatment with ixekizumab may interrupt the IL-17/IL-23 axis^35,36^. The subsequent decrease in JunB and other factors may contribute to maintenance of Th17 pathogenicity^37,38^, leading to rapid declines in important cytokines such as IL-17 that can synergize with IL-19 in keratinocytes^39^. As IL-17-induced IL-19 production by epidermal cells is considered one of the contributors for keratinocyte proliferation using human skin models^35^, it is very intriguing that rapid normalization of IL-19 levels correlates with subsequent decreases in epidermal thickness following treatment with an anti-IL-17A antibody such as ixekizumab^35^. It will be interesting to define further the role of IL-19 as a marker for psoriatic inflammation, given the ability of IL-19 and IL-19-activated macrophage-conditioned medium to mediate angiogenic reactions^40^ and activated macrophage-mediated vascular inflammation^33^.

While it has been traditionally assumed that higher PASI scores assessed by visual examination of the skin in patients with psoriasis correlate with higher levels of inflammation^41,42^, a recent report suggested that the level of inflammation might be even higher in skin lesions with limited surface area^43^. Even mild psoriasis has been characterized by increased IL-17 expression and infiltrating T cells in the skin lesions^43^. These observations, combined with our data demonstrating that some psoriasis, genital psoriasis, and psoriatic arthritis patients with relatively low PASI scores have elevated circulating IL-19, further underscore the need for an objective measure of psoriatic disease involvement.

This is particularly relevant in light of a recent publication that demonstrated that in patients with psoriasis, IL-17–producing T cell clones resided even in psoriatic skin lesions that appeared to have resolved clinically^44^. This finding suggests that these cells may constitute the pathogenic T cells that can re-initiate skin relapses, indicating that lasting control of psoriatic skin disease will require suppression of these resident T cell populations^44^. If this is indeed the case, then the ability to detect increases in serum IL-19 that precede relapse of skin disease may be especially important. Similarly, it has been established that in patients with atopic dermatitis, the non-lesional skin is not entirely normal or healthy, supporting the idea that the skin’s immune system is hyper-stimulated^45,46^. Our IL-19 data raise the possibility that “mild” skin disease may not truly reflect the overall systemic immune activation status of any given patient. In addition, the recognition that IL-19 may reflect epidermal keratinocyte biology^47^ makes our ability to measure circulating IL-19 particularly relevant.

In light of our findings, we contemplated the mechanisms that could account for the potential role of IL-19 as a biomarker for psoriasis, atopic dermatitis, and possibly other epithelial-based diseases. The IL-19 promoter is complex and contains multiple binding sites for nuclear factor κB (NFκB), STAT6, and keratinocyte growth factor (KGF)^48–50^. Two main inducers of IL-19 mRNA in keratinocytes are IL-17 and TNFα; both of these cytokines signal through the NFκB pathway^48,51^. These are likely the dominant drivers in psoriasis, particularly IL-17 secreted from Th17 cells (which are in turn positively regulated by IL-23)^52^. However, IL-19 mRNA expression is also increased by the Th2 cytokines IL-4 and IL-13, which work primarily through STAT6 to drive IL-19 expression^53–55^. These are likely the dominant drivers in atopic dermatitis and possibly asthma as well. Importantly, IL-17 and IL-4/IL-13 act synergistically to increase IL-19 expression through the combined effects of NFκB and STAT6, respectively, on the IL-19 promoter^53–55^.

In keratinocytes, secreted IL-19 acts via its canonical receptor (IL20Rα/IL20Rβ)^9^. In addition, IL-19 can also signal in several different types of immune cells (although the mechanism is not completely elucidated, since immune cells do not appear to express IL20Rα)^48–50^. IL-19 may stimulate CD8+ T cells and fibroblasts to release KGF, which can then further upregulate IL-19 in keratinocytes^56,57^. In addition, IL-19 can signal Th2 cells to release IL-4 and IL-13^58^. These cytokines bind to the IL-4 receptor on keratinocytes to activate the JAK/STAT pathway to increase STAT6, which then amplifies upregulation of IL-19^53–55^. IL-19 can also directly stimulate monocytes to release TNFα, which signals through NFκB^51,59^. The increased NFκB then synergistically combines with the increased STAT6 to increase further expression of IL-19^53–55^. Finally, IL-19 can amplify many of the effects of IL-17 on keratinocytes, including upregulation of β-defensins, IL-23p19, and Th17 and neutrophil-attracting chemokines^39^. Thus, IL-19 may be positioned as a marker at the junction where the Th17 and Th2 pathways converge^48–50,60^.

Because IL-19 is expressed by activated keratinocytes and macrophages, IL-19 produced locally can make its way into the systemic circulation, thereby allowing a serum measurement to provide a unique and objective window through which to view the true, underlying severity of disease. This is particularly important because as the therapeutic revolution in psoriasis and atopic dermatitis continues, clinicians will likely be looking for reliable and objective indicators of overall disease, especially as they contemplate transitioning patients from topical to systemic treatments. Use of a circulating IL-19 assay may also improve our understanding of under what conditions complete resolution of these lifelong diseases might be possible.

Thus, a serum IL-19 assay might be considered not only for replacing or augmenting visual assessment tools used to categorize psoriasis severity, but also for documenting the timing of the therapeutic responses, assessing the degree of efficacy, and serving as a harbinger of relapse when treatments need to be discontinued. Reliable quantitation of circulating IL-19 may provide healthcare professionals with an objective, biochemical assessment for evaluating disease activity in psoriasis patients that avoids inter-observer variability from visual examinations^41,42^. In addition, healthcare professionals currently performing EASI assessments for atopic dermatitis patients may be similarly aided by such a method.

## Supplementary information

Supplementary File

## Data Availability

Eli Lilly and Company provides access to relevant anonymized patient level data from studies on approved medicines and indications as defined by the sponsor specific information on www.clinicalstudydatarequest.com. For details on submitting a request, see the instructions provided at www.clinicalstudydatarequest.com.
