# Supplementary material for: Assessment and Clinical Relevance of Serum IL-19 Levels in Psoriasis and Atopic Dermatitis Using a Sensitive and Specific Novel Immunoassay
Source: Sci Rep. 2019 Mar 26;9:5211. doi: 10.1038/s41598-019-41609-z (PMC6435799; doi:10.1038/s41598-019-41609-z)
Supplement: Supplementary file 1 — Supplementary File [file 41598_2019_41609_MOESM1_ESM.pdf]

Supplementary File

Assessment and Clinical Relevance of Serum IL-19 Levels in Psoriasis and Atopic Dermatitis Using a Sensitive and Specific Novel Immunoassay

Robert J. Konrad, Richard E. Higgs, George H. Rodgers, Wenyu Ming, Yue-Wei Qian, Nicoletta Bivi, Justin K. Mack, Robert W. Siegel, and Brian J. Nickoloff

From the Lilly Research Laboratories, Eli Lilly and Company, Indianapolis, IN 46285, USA

Supplementary Methods

Expression and purification of human recombinant IL-19 – The codon-optimized nucleotide sequences encoding mature IL-19 were inserted into pET21a (Novagen) vector. Bacteria BL21(DE3) (Novagen) was used as expression host, and the induction of protein expression was carried out in 2X TY media with 1 mM IPTG at 37ºC for 5 hours. Cells were harvested and stored at -80ºC for subsequent protein refolding and purification, which was conducted at 4ºC. Frozen cell pellets were lysed by incubation with stirring in 40 mL lysis buffer (50 mM Tris-HCl pH 8.0, 250 mM NaCl, 1 mM EDTA, 0.2% Triton X-100, 0.1 mg/mL lysozyme, 5 U/mL benzonase, Pierce protease inhibitor tablets) per liter cell pellet and sonication. Cell lysates were centrifuged at 10,000 rpm for 20 minutes. Pellets were washed once with buffer A (50 mM Tris-HCl pH 8.0, 250 mM NaCl, 1 mM EDTA) containing 0.2% Triton X-100 and three times with buffer A by re-suspension and centrifugation. Purified inclusion bodies were solubilized (50 mM Tris-HCl, pH 8.0, 6M urea, 200 mM NaCl, 20 mM DTT), incubated for 2 hours at room temperature, and centrifuged at 15,000 rpm for 20 minutes. The protein concentration of the supernatant was adjusted to 2 mg/mL with the same buffer. Protein refolding was performed by 1:20 dilution into

refolding buffer (50 mM Tris-HCl pH 8.0, 200 mM NaCl, 1 M urea, 0.5 mM reduced glutathione and 0.5 mM oxidized glutathione) at 4ºC for 48 hours. Refolded protein was concentrated, loaded onto a HiLoad 26/600 Superdex 75 column, and eluted with storage buffer (20 mM HEPES, pH 7.0, 150 mM NaCl, 10% glycerol). Fractions containing refolded IL-19 were pooled, and the final protein concentration was determined by absorbance (UV_{280}). Recombinant IL-19 protein was aliquoted and stored at -80ºC prior to use.

Generation, purification, selection, and labeling of murine monoclonal anti-human IL-19 antibodies – All experimental mouse protocols were approved by the Institutional Animal Care and Use Committee of A&G Pharmaceutical, Inc. (Columbia, MD, USA) and were conducted in accordance with their relative regulations and guidelines, with periodic review by Eli Lilly Veterinary resources. Anti-IL-19 hybridoma clones were obtained by fusing splenocytes from mice immunized with recombinant human IL-19 (prepared as described above). Clones were screened for non-overlapping epitopes and affinities determined after titrations of 0.37-270 nM of IL-19 protein using Octet Red96 (Pall ForteBio, Menlo Park, CA, USA) by global fitting of a 1:1 binding model using Data Analysis v9.0 software. The antigen-specific variable heavy (VH) and variable light (VL) gene sequences were determined from clones of interest. Sequences were transferred into separate murine IgG_1 and IgK constant region expression vectors for recombinant antibody production and subsequent purification using protein A chromatography after large-scale transient transfection in HEK293 cells. Affinity maturation of the parent capture antibody was performed as previously described[1,2] with minor modifications. Briefly, VH and VL domains were combined to construct a single-chain Fragment variable (scFv) through single overlap extension PCR. The VH and VL domains were ligated into display vector, and mutagenic libraries of the VH

and VL complementarity determining regions were constructed by spiked mutagenesis using degenerate oligonucleotides. Libraries were screened for improved binding after iterative rounds of selection using decreasing amounts of biotinylated recombinant IL-19 by fluorescence-assisted cell sorting on a FACS Aria cell sorter (BD Biosciences, San Jose, CA, USA). Sequences of clones with enriched binding were determined and placed into expression vectors. Two monoclonal antibodies directed against separate IL-19 epitopes were selected for labeling. One milligram of each antibody was biotinylated using a Pierce kit and ruthenium-labeled using a MesoScale Discovery (MSD) kit for electrochemiluminescent (ECL) detection. Antibodies were evaluated using Matrix-Assisted Laser Desorption - Ionization-Time of Flight Mass Spectrometry (MALDI-TOF) to ensure that suitable labeling had occurred. Labeled antibodies were diluted in 50% glycerol and stored at -20°C prior to use.

IL-19 immunoassay – A human IL-19 MSD sandwich immunoassay was developed using reagents described above. Streptavidin-coated MSD plates were washed three times with TBST (Tris buffered saline containing 10 mmol/L Tris pH 7.40, 150 mmol/L NaCl with 1 mL Tween 20/L). Plates were blocked with TBST plus 1% bovine serum albumin for 1 hour at room temperature. After aspiration and washing with TBST, wells were incubated with biotinylated anti-IL-19 capture antibody (1 mg/L) for 1 hour. Following aspiration and washing with TBST, 50 µL of recombinant human IL-19 standards (serially diluted IL-19 recombinant protein ranging from 0-50,000 pg/mL), were added to the wells in assay buffer (50 mmol/L HEPES, pH 7.40, 150 mmol/L NaCl, 10 mL/L Triton X-100, 5 mmol/L EDTA, and 5 mmol/L EGTA). Serum samples were diluted 1:4 in assay buffer and added to their respective wells, and the ELISA plate was incubated overnight at 4°C. After aspiration, wells were washed three times with TBST, and 50 µL of

ruthenium-labeled anti-IL-19 detection antibody (0.5 mg/L) was added for a 1-hour incubation at room temperature. Following aspiration, wells were washed three times with TBST, and 150 μL of 2X MSD read buffer was added. Ruthenium ECL in the wells was detected using a MSD plate reader. Specificity of the assay for IL-19 was confirmed by evaluating cross-reactivity to other cytokines, including additional members of the IL-10 cytokine family. Cross-reactivity was assessed using recombinant human TNFα (produced internally), IL-17 (R&D Systems, Minneapolis, MN, USA), and the following members of the IL-10 family of cytokines: IL-10, IL-20, and IL-24 (Novus Biologicals, Littleton, CO, USA), IL-26 monomer and IL-26 dimer (R&D Systems, Minneapolis, MN, USA), and IL-22 (produced internally).

Supplementary Results

Monoclonal anti-IL-19 antibodies were developed for use in a sandwich immunoassay to measure IL-19 in human serum (Figures S1A and S1B). Kinetic analysis of the antibodies with non-overlapping epitopes indicated poor affinity with one of the antibodies (Figure S1A) precluding our ability to measure endogenous normal levels of IL-19. Subsequent *in vitro* affinity optimization resulted in an antibody with greatly improved binding kinetics (Figure S1C). The optimized antibody (which became the capture antibody for the assay) had an equilibrium affinity constant (K_D) of 5.76 x 10^{-11} M, which was approximately 13,000-fold better than the parental antibody.

The optimal pairing used biotinylated capture antibody and ruthenium-labeled detection antibody on a streptavidin-coated MSD plate. Figure S1D shows the consistency of the assay across ten different runs. The assay showed acceptable dynamic range, background, sensitivity, and dilutional linearity. Inter-assay precision results (CVs) were less than 10% at all concentrations tested, and recoveries at all concentrations tested were 80-120%, even after six freeze-thaw cycles. To ensure that IL-19 was not altered during the clotting process, we collected matching serum and EDTA plasma samples from healthy donors. All serum results were within 15% of EDTA plasma values. Finally, IL-17, TNFα, and members of the IL-10 family (IL-10, IL-20, IL-22, IL-24, and IL-26) demonstrated no cross-reactivity on the assay at concentrations as high as 25,000 pg/mL, confirming specificity for IL-19 (data not shown).

Supplementary Figures

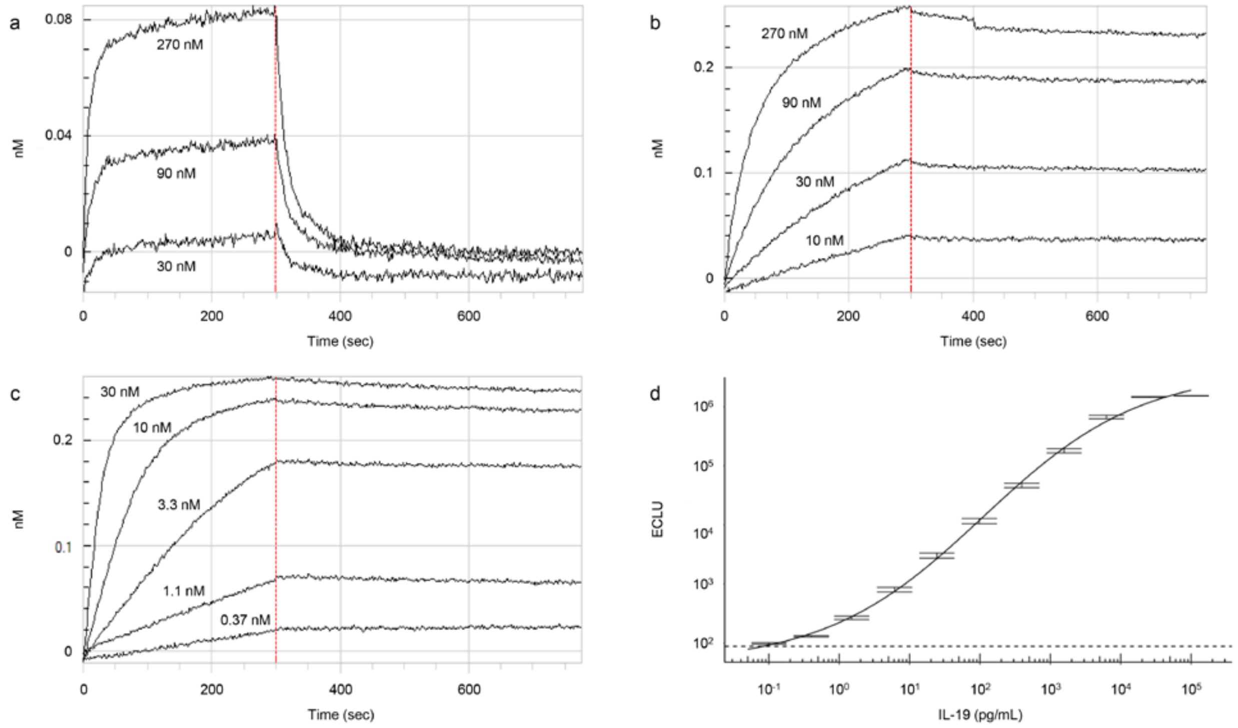

Figure S1: Development of capture and detection antibodies and the IL-19 immunoassay

A: Bio-layer interferometry sensorgram of parental capture antibody titrated with IL-19, with binding data from each concentration shown. Time is on the X-axis, and wavelength shift on the Y-axis. The vertical line marks the end of the association phase.

B: Interferometry sensorgram of detection antibody titrated with IL-19.

C: Interferometry sensorgram of optimized capture antibody titrated with IL-19.

D: A sandwich immunoassay was developed using the antibodies in Figures S1B and S1C to measure human serum IL-19. Recombinant human IL-19 was for a calibration curve starting at 50,000 pg/mL with serial dilutions. Each curve point is shown as the 95% confidence interval from 10 separate runs. The dashed horizontal line indicates the mean level of the zero calibrator.

Supplementary References

1. Siegel, R.W. Antibody affinity optimization using yeast cell surface display. *Methods Mol Biol* **504**, 351-383 (2009).
2. Van Deventer, J.A. & Wittrup, K.D. Yeast surface display for antibody isolation: library construction, library screening, and affinity maturation. *Methods Mol Biol* **1131**, 151-181 (2014).
